# Genome Sequences of Ilzat and Eleri, Two Phages Isolated Using Microbacterium foliorum NRRL B-24224

**DOI:** 10.1128/genomeA.00144-18

**Published:** 2018-04-12

**Authors:** Ilzat Ali, Acacia Eleri Jones, Aleem Mohamed, Viknesh Sivanathan

**Affiliations:** a Department of Chemistry and Biochemistry, University of Maryland Baltimore County, Baltimore, Maryland, USA; b Department of Biological Sciences, University of Maryland Baltimore County, Baltimore, Maryland, USA; c The Cathedral School, Llandaf, United Kingdom; d Science Education, Howard Hughes Medical Institute, Chevy Chase, Maryland, USA

## Abstract

Bacteriophages Ilzat and Eleri are newly isolated Siphoviridae infecting Microbacterium foliorum NRRL B-24224. The phage genomes are similar in length, G+C content, and architecture and share 62.9% nucleotide sequence identity.

## GENOME ANNOUNCEMENT

We report here the genome sequences of two actinobacteriophages, Ilzat and Eleri, isolated from soil collected in Aquilla, TX, and College Park, MD, respectively, using Microbacterium foliorum NRRL B-24224 as the host. The phages were isolated by enrichment at 30°C on peptone-yeast extract-calcium (PYCa) medium supplemented with 0.1% dextrose (1, 2). Transmission electron microscopy revealed Ilzat and Eleri to be members of the Siphoviridae family, with flexible tails of 112 nm and 120 nm in length and isometric capsids of 46 nm and 51 nm in diameter, respectively. When plated using top agar overlay, both phages form plaques with clear centers and turbid edges.

Double-stranded DNA isolated from both phages was sequenced using an Illumina MiSeq 150-bp single-end run. Trimmed reads were assembled using Newbler, yielding circularly permuted genomes of similar length and G+C content, with 41,525 bp and 63.5% for Ilzat and 40,366 bp and 62.0% for Eleri, respectively. The G+C contents of both phages are lower than that of the isolation host (68.7%; GenBank accession no. JYIU00000000).

The genomes were annotated using DNA Master (http://cobamide2.bio.pitt.edu/), Glimmer (3), GeneMark (4), BLASTP (5), HHpred (6), and Phamerator (7). A total of 62 and 63 protein-coding genes were identified in Ilzat and Eleri, respectively. The two phages share similar genome architectures and synteny, with DNA packaging, virion structure and assembly, and lysis genes occupying the left half of the genome and transcribed rightwards and DNA metabolism genes occupying the right half of the genome and transcribed leftwards, with the exception of the rightmost 1 or 2 genes that are transcribed rightwards. Using EMBOSS Stretcher, the phages share 62.9% average nucleotide identity, with segments of high identity clustered primarily in the leftmost three-quarters of the genomes (7). Both phages belong to cluster EA, with Ilzat and Eleri separated into subclusters EA1 and EA2, respectively.

Functions can be assigned to 23 genes, 22 of which are present in both phages. Of these, the majority share >60% amino acid sequence similarity between the two phages and include terminase and portal proteins, major capsid and capsid maturation proteins, several tail proteins and tail assembly chaperones, DNA recombinase (RecA), helicase, and DNA polymerase (Pol I) proteins, as well as ThyX thymidylate synthase. Within the region containing DNA metabolism genes, both phages encode a hypothetical protein (Ilzat gene product 35 [gp35] and Eleri gp34) with stretches of glutamate and aspartate residues. D/E repeats are implicated in DNA/RNA functions through charge mimicry of nucleic acids (8). The remaining shared gene products have lower amino acid sequence similarities. These include lysin A (55.7%) and holin (56.9%), which flank a membrane protein of unknown function (59.0%), as well as a MazG nucleotide pyrophosphohydrolase (34.5%), which, in mycobacteria, safeguards against DNA mutations under oxidative stress (9). Ilzat encodes a phosphoesterase that is not encoded in Eleri.

### Accession number(s).

The phage genome sequences are available at GenBank with the accession no. MG839029 (Ilzat) and MG839027 (Eleri).
